# Transoral thyroid surgery vestibular approach: is there an increased risk of surgical site infections?

**DOI:** 10.1007/s13304-021-01191-4

**Published:** 2021-10-20

**Authors:** Elias Karakas, Günther Klein, Linda Michlmayr, Martin Schardey, Stefan Schopf, The Endoscopic Thyroid and Parathyroid Surgery Study Group

**Affiliations:** 1 Department of General-, Abdominal- and Endocrine Surgery, Hospital Maria Hilf, Alexianer GmbH, 470805 Krefeld, Germany; 2 Department of General Surgery, Landesklinikum Wiener Neustadt, Wiener Neustadt, Austria; 3 Department of General-, Abdominal and Vascular Surgery, Hospital Agatharied, Hausham, Germany; 4 Department of General-, Abdominal- and Endocrine Surgery, RoMed Hospital, Bad Aibling, Germany

**Keywords:** Transoral surgery, Thyroid, Infection rate, Prevention

## Abstract

Transoral endoscopic thyroidectomy vestibular approach (TOETVA) is an upcoming surgical technique with the aim to optimize cosmetic outcome avoiding visible scars in the neck. However, the transoral access bears the risk of contamination and microbial allocation from the mouth into the thyroid region. Therefore, some authors recommend extended antibiotic therapy up to 7 days after surgery. Our aim was to evaluate infection rates and parameters before and after transoral surgery and to suggest a viable and safe routine in transoral surgery. Prospectively collected data of patients who were eligible for transoral surgery in Austria and Germany between June 2017 and July 2020 were retrospectively evaluated focussing on clinical and laboratory infection signs pre and postoperatively. White blood cell count (WBC) and C-reactive protein levels (CRP) were estimated before and after surgery. Patients` characteristics, surgical outcome and complications were also determined and compared to the current results reported in the literature. 113 transoral operations were performed in 108 patients. In 37 of 108 (36%) patients an additional retroauricular incision in the hairline and in two patients a submental skin incision was performed to extract thyroid specimen of more than 40 ml. Intravenous antibiotic prophylaxis and enoral mucosal disinfection were used in all patients before surgery. WBC and CRP levels were available in 75 patients. Median WBC was 5800/µl (range 3500–10,500/µl) before and significantly higher (median 8900/µl, range 4500–18,800 µl; *p* < 0.01) at day one after surgery. WBC returned to normal range (4500–11,500/µl) in all patients within the first 7 days postoperatively (median 5300/µl, range 3400–8700/µl). CRP levels were normal before (< 0.5 mg/dl) and slightly elevated within the first two days after surgery (Median 2.0 mg/dl, range 0.5–6.4 mg/dl, n.s.). In one patient oral antibiotic therapy was necessary due to transient erythema in the chin region which occurred 10 days after surgery and resolved completely without surgical intervention. Despite a transient increase in WBC transoral thyroid and parathyroid surgery via the vestibular approach does not seem to be associated with a significant number of wound infections in our patients. Intravenous antibiotic prophylaxis and enoral mucosal disinfection might be reasonable procedures to avoid microbial allocation from the mouth into the thyroid region. However, further investigations are required to finally estimate the need of antibiotics in transoral surgery.

## Introduction

Conventional thyroid and parathyroid surgery are clean with rates of surgical site infections (SSI) reported to be between 0.5 and 3% at most [1–5].

Specific risk factors have been identified to be associated with a higher rate of SSI in thyroid surgery like central lymph node dissection with thyroidectomy compared with thyroidectomy alone [3], total thyroidectomy compared with lobectomy [4] or obesity [6]. In addition, smoking and diabetes are reported risk factors for SSI in other surgical fields and might increase the risk of SSI also in thyroid surgery [7, 8].

Over the past two decades various techniques of minimally invasive thyroidectomy like transcervical video assisted surgery, transaxillary, breast or postauricular (EndoCATS) have been developed because many patients were concerned about the scar in the visible part of the neck. Though, these techniques are associated with new, intervention specific complications, none of these procedures is susceptible to be associated with infection rates higher than conventional thyroid surgery [9, 10].

Transoral endoscopic thyroid surgery via the vestibular approach (TOETVA), representing the newest and currently most considered extracervical approach, is performed via mucosal incisions in the vestibulum oris. TOETVA as well as its modifications, like the combined transoral and retroauricular approach (TOVARA) that allows for retrieving specimen of more than 40 ml volume show excellent cosmetical results by avoiding visible scars with low complication rates [11, 12].

However, the transoral access might be associated with new hitherto unknown complications, like skin lesions in the chin area or the potential of contamination and microbial allocation from the mouth into the thyroid region.

Therefore, some authors recommend prophylactic as well as postoperative antibiotic therapy for 3–7 days, representing a marked difference to conventional thyroid surgery [12, 13].

After implementation, modification, and thorough evaluation of TOETVA and TOVARA in Germany and Austria our aim was to evaluate the risk of surgical site infections with preoperative intravenous antibiotic prophylaxis and enoral mucosal disinfection.

## Patients and methods

We retrospectively evaluated all prospectively recorded data of patients who underwent TOETVA with or without an additional retroauricular incision (Transoral Endoscopic thyroid surgery vestibular and retroauricular access, TOVARA) from June 2017 through July 2020. All operations were performed by or under supervision of three surgeons (S.S., G.K., E.K.). All patients who underwent TOETVA and TOVARA were included. Exclusion criteria included previous neck and chin surgery, substernal goitre, clinically evident lateral neck lymph node metastasis or distance metastasis, suspicious invasive growth of cervical mass, preexisting enoral infection, like herpes, or recurrent laryngeal nerve palsy and oral anticoagulant intake.

Written informed consent written informed consent was obtained in all patients who underwent transoral surgery also regarding collection, analysis, storage and/or reuse of data. Data were anonymously collected, and the study has been conducted in accordance with the Declaration of Helsinki.

In addition, approval to conduct this retrospective study was obtained from the Ethical committee of the Ludwig Maximilians-University, Munich Germany (Reference Number: 21-0916).

Transoral surgery was chosen based on patients’ preferences, size of the tumour, and the nature of the disease. Preoperative evaluation included thyroid function tests, and thyroid ultrasonography. Scintigraphy and fine-needle aspiration cytologic testing were also used in some but not all patients. No routine dental examination was required.

### Surgical technique

Transoral surgery was performed by placing the patient in a supine position with slight neck extension under orotracheal intubation. Amoxicillin–Sulbactam acid, 3 g, was administered at least 30 min before incision. Three laparoscopic ports, a 5–12 mm port at midline and two 3–5 mm ports were inserted straight under the lower lip at the oral vestibular area lateral between the canine and first premolar teeth. According to the size of the midline-port a standard 5 mm or 10 mm 30° rigid laparoscope was used. The working space was created down to the sternal notch and lateral until the sternocleidomastoid muscles. The strap muscles were separated in the midline and retracted laterally by a transcutaneous suture (2.0 PDS) to widen the working space together with a low pressure (6–8 mmHg) CO_2_ gas insufflation to expose the thyroid and trachea. The thyroid isthmus was divided first. Next, the superior pole was dissected, and the branches of the upper pole vessels were divided using a thermal device. Upper parathyroid and lower parathyroid glands were routinely identified and preserved. Due to the top-down visualization the recurrent laryngeal nerve (RLN) was identified at its insertion to the larynx and visualized down and parallel to the trachea. After resection thyroid lobe specimen was placed in an extraction bag and either retrieved through the central incision or an additional retroauricular subcutaneous access. For total thyroidectomy, the operation was performed in the same manner on the contralateral side, but only one retroauricular access was created on one side. No drain was placed, and a pressure dressing was placed around the chin and upper neck for 24 h some but not all patients.

### Postoperative management

All patients could drink after complete recovery from anaesthesia on the day of surgery and could eat and drink as usual on the following day. None of the operations were performed as outpatient procedures. Intravenous antibiotics were not routinely given postoperatively. Postoperative analgesic medication included anti-inflammatory drugs (ibuprofen, diclofenac, or metamizole dipyrone) was the same as in open thyroid surgery. Few patients required additional intravenous analgesia in the ward. All patients were evaluated by laryngoscopy two days postoperatively. Follow-up examinations were scheduled at 1 week, 1 month, 6 months, and 1 year.

### Outcome

Medical records were reviewed for patient characteristics and outcomes, including incision to closure time, estimated intraoperative blood loss, and WBC and CRP levels. Complications were identified. RLN injury was defined as impaired movement of one or both vocal cords on laryngoscopy. Permanent RLN injury was defined as an injury that did not recover within 6 months. Seroma, hematoma, and signs of local infection including interventions if applicable were recorded. Hypoparathyroidism was defined as a parathyroid hormone level less than 12 pg/mL (normal range 12–88 pg/ml) 24 h postoperatively. Permanent hypoparathyroidism was defined as no recovery within 6 months. Paraesthesia of the lower lip was defined as mental nerve palsy. Hematoma was defined as obvious postoperative livid discoloration of the skin, while severe hematoma or bleeding was defined as neck swelling and required reoperation. Infection was defined as postoperative local abscess, high-grade fever with evidence of systemic bacterial spread that required antibiotic and/or surgical treatment.

### Statistical analysis

Baseline characteristics were compared using the Fisher exact test for categorical variables and the independent, 2-tailed, paired *t* test for continuous variables. *p* < 0.05 was statistically significant. Statistical analysis was performed with SPSS statistical software, version 20.0 (IBM Inc). All values were expressed as median and mean (SD) for continuous variables and number (percentage) for categorical variables.

## Results

### Primary outcome—local or systemic inflammation

One hundred-thirteen transoral operations were performed in 108 patients. In 37 of 108 (36%) patients an additional retroauricular incision in the hairline and in two patients a submental skin incision was performed to extract thyroid specimen of more than 40 ml. All patients were included because the transoral approach was established in all being at risk for contamination and microbial allocation from the mouth into the thyroid region.

According to requirements recommended by Anuwong [12] intravenous antibiotic prophylaxis and enoral mucosal disinfection was used in all patients before surgery. In contrast to these recommendations, antibiotics were given preoperatively only, and prophylaxis was not extended routinely for 1 week. WBC and CRP levels were available in 75 patients (69%, Table 1). Median WBC was 5800/µl (range 3500–10,500/µl) before and significantly higher (median 8900/µl, range 4500–18800 µl; *p* < 0.01) at day one after surgery. WBC returned to normal range (4500–11,500/µl) in all patients within the first 7 days postoperatively (median 5300/µl, range 3400–8700/µl). CRP levels were normal before (< 0.5 mg/dl) and slightly elevated within the first two days after surgery (Median 2.0 mg/dl range 0.5–6.4 mg/dl, n.s.).

**Table 1:**
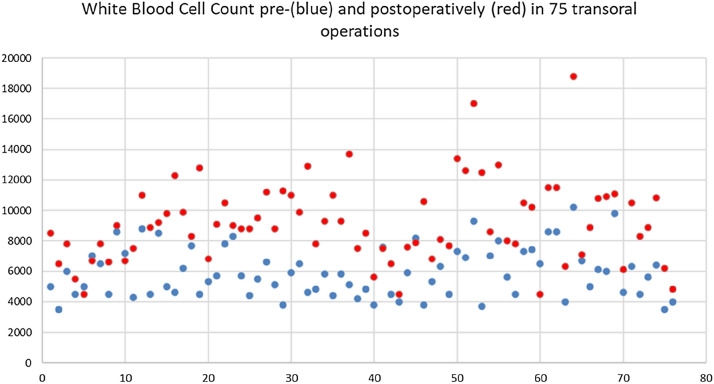
White blood cell count pre- and postoperatively in 75 patients with transoral surgery

Follow-up data were available in all patients 6 weeks after surgery. In one patient oral antibiotic therapy was necessary due to transient erythema in the chin region which occurred 10 days after surgery and resolved completely without surgical intervention.

In 90 patients clinical follow-up investigation was performed 1 year after surgery with excellent cosmetic outcome and no clinical signs of infection.

Only one of the 108 patients who underwent TOETVA (mean age, 49.1 (+ 11.6) years; range: 23–77 years; 103 (95.3%) female, 5 (4.7%) male) was converted to open surgery because of the huge size of the thyroid specimen (volume: 130 ml, weight: 98 g, thyroid lobe larger than 10 cm) in an adipose female patient with a short neck. Despite the high tissue trauma and significant extension of the operation time no infection occurred in this patient.

### Secondary outcome—surgical outcome and complications

In 54 (47.8%) patients hemithyroidectomy was performed, and 52 patients (46.0%) underwent total thyroidectomy. In 4 patients (3.5%) only isthmic resection was necessary, while two operations (1.8%) were done because of a solitary parathyroid adenoma. In one patient (0.9%) a thyroglossal cyst was removed transorally. Ten patients (9.3%) had Graves` disease, in 10 patients papillary carcinoma (6 microcarcinomas, 3 stage one and one stage 2 carcinomas) was detected. One patient suffered from follicular thyroid cancer. The specimen size was 34 + 26 ml (range, 4–130 ml), with an operative time of 198 (+ 67) minutes (hemithyroidectomy, 179 (+ 59) minutes; total thyroidectomy, 226 (+ 65) minutes). Total estimated blood loss was negligible (30.8 (+ 25.1)ml. The overall VAS pain score was 1.5 (+ 1.2), and the VAS pain scores were 2.0 (+ 1.8), 1.3 (+ 1.1), and 0.5 (+ 0.6) for the first 3 days. Seven patients (4.5%) had transient RLN palsy; all cases resolved within 6 months. One patient suffered from permanent RLNP following surgery due to Graves` disease. In this female patient the voice handicap score was 4 out of 5 without signs of dyspnoea, but normal voice without voice fatigue. Therapy was completed by radioiodine 6 months after surgery.

Ten patients (9.3%) had transient hypoparathyroidism; none had permanent hypoparathyroidism. One patient showed signs of postoperative bleeding directly after surgery within the same anaesthesia and successful transoral reoperation was done immediately. Two patients (1.9%) had slight but permanent mental nerve injury. In one patient a small (0.2 × 0.2 cm) but apparent skin lesion in the chin area occurred and remained as a small but permanent scar. No patient had enoral or retroauricular wound infection (Table 2). When we performed the analysis by intention to treat and included one patient who was converted to open thyroid surgery, the results remained the same.

**Table 2:** Clinical data, peri- and postoperative outcome of patients who underwent TOETVA and TOVARA

	*n*	%
Number of patients	108	
Number of operations	113	
Patients` age (years, median, range)	49.1 (23–77)	
Male	5	5
Female	103	95
Thyroid lobe volume < 40 ml	81	76
Thyroid lobe volume > 40 ml	25	24
Operation time (min, median, range)	198 (90–420)	
Procedure		
Hemithyroidectomy	54	48
Total thyroidectomy*	52	46
Parathyroid resection, primary hyperparathyroidism	2	2
Isthmic resection	4	3
Thyroglossal cyst resection	1	1
Postoperative malignancy (papillary and follicular thyroid cancer)**	11	10
Graves' disease**	10	9
Nerves at risk	157	
Transient RLNP	7	4.5
Permanent RLNP	1	0.6
Transient hypoparathyroidism	10	9
Permanent hypoparathyroidism	0	0
Permanent mental nerve dysfunction	2	3
Postoperative seroma	10	9
Postoperative bleeding***	1	0.6
Small skin lesion chin area	1	0.6
Wound infection	0	0

*Due to multinodular goitre, Graves` disease or completion thyroidectomy

**Included in “procedure”

***Transoral reoperation within the same anaesthesia

## Discussion

The transoral endoscopic thyroidectomy vestibular approach (TOETVA) has been successfully established in many specialized centres all over the world. Current data available show few complications and excellent results especially regarding cosmesis. However, debate remains about whether transoral surgery is appropriate and potentially associated with an increased risk of surgical site infection. The transoral access is categorized as a clean-contaminated wound and bears the risk of contamination and microbial allocation from the mouth into the thyroid region. None of the data regarding transoral surgery published point to an increased infection or contamination rate [13]. In addition, investigations, and operations in pigs via an enoral para- or sublingual approach were inconspicuous regarding infection or contamination of the operating field [14]. Microbiological swabs taken 14 days after surgery from the operation field were clean and no clinical signs of wound infection were detected. Our observations are in line with practical clinical experiences in head and neck surgery, oral and maxillofacial surgery and transoral spine surgery to the palatopharyngeal junction, transoral resection of retropharyngeal lymph node metastases, and transoral removal of submandibular glands in humans with negligible contamination rates of the access route and the operative field [15–19].

In contrast to other groups our patients received single shot antibiotic prophylaxis instead of perioperative antibiotic therapy for up to 7 days postoperatively and enoral mucosal disinfection only, with inconspicuous postoperative outcome in 104/105 patients [12, 13, 22]. Only one female patient was treated with oral antibiotics for 1 week due to transient erythema in the chin region which occurred 10 days after transoral hemithyroidectomy due to a benign solitary nodule and resolved completely without intervention. Operation time was 174 min without complications. Compared to rates of surgical site infections in conventional thyroid surgery reported to be between 0.5 and 3% at most, our rate is at the lower bound (1/108 patients 0.9%) despite the vestibular “clean-contaminated” access.

Routine determination of WBC or CRP before and after conventional thyroid surgery is uncommon. We were therefore not able to compare laboratory parameters of transoral and conventional thyroid procedures. We observed a significant increase in WBC count within the first 24 h after transoral surgery (*p* < 0.01). Though increase was obvious WBC count remained within normal range in 67/75 patients. Only eight patients showed transient leucocytosis which declined into normal range within 7 days postoperatively. Two of these patients underwent transoral completion thyroidectomy within the same hospital stay due to thyroid cancer. Leucocytosis was not correlated to clinical signs of infection. Previous studies on complications after conventional thyroid surgery showed that patients who underwent lymph node dissection, who were reoperated due to bleeding and who had a drain postoperatively had increased risk of SSI [20]. The authors concede that the number of transoral procedures performed and the operative spectrum in transoral surgery is rather small because patients eligible for transoral surgery are highly selected. Extensive lymphadenectomy or redo-surgery was not performed. However, current data suggest that careful selection of patients might be an important key to avoid unacceptable results after transoral surgery. In addition, emergency reoperation due to postoperative bleeding fortunately was not necessary to date.

Despite long operation times duration of operation reported as an independent risk factor for SSI in thyroid surgery by Burres et al. [21] does not lead to an increase in SSI in transoral surgery. This might be due to the fact, that single shot antibiotic administration and use of mucosal disinfectant was routinely used in our patients. In addition, patients with obvious local peri- and enoral signs of infection like herpes labialis were excluded from transoral surgery. In contrast to Asian patients with commonly lower body mass indexes (calculated as weight in kilogrammes divided by height in metres squared, BMI) thyroid surgery is more often performed in obese patients in Europe. In our series, there were 40 patients with a BMI > 28. We consent to data reported by Anuwong et al. [13] suggesting that TOETVA can be performed safely in obese patients and without increased SSI.

## Limitations

Our study has some limitations. Because WBC and CRP levels were not available from patients with OT a propensity score analysis was not possible. Patients were highly selected in three highly specialized centres and the total number of patients´ reports is rather low. In addition, infection parameters were available in only 75/108 patients and not in all. WBC and PCR were estimated as biochemical surrogates of infection but were retrieved only in 75 patients over 108 (69%). Although this is a substantial loss of information at follow-up, data suggest that transoral surgery might not be associated with an increase in surgical site infections. Only one out of 108 patients showed signs of inflammation 10 days after surgery and in none of the 90 patients who underwent clinical reevaluation after 12 months clinical signs of inflammation were observed.

Most patients in this study had benign lesions. Multicenter, multicountry studies, as well as prospective studies with long-term follow-up, are needed to confirm the safety and effectiveness of transoral surgery, especially in patients with thyroid cancer.

## Conclusion

Transoral thyroid and parathyroid access using the TOETVA technique with or without an additional retroauricular approach is also feasible and safe. As infection rates seem to be at least as low as in conventional open thyroid surgery, infection does not seem to be a specific problem of transoral thyroid surgery. Single shot antibiotic application before surgery instead of extended antibiotic therapy might be sufficient as it is used in many institutions also for open thyroid surgery.
